# Effects of Clinical Use on the Mechanical Properties of Bio-Active^®^ (BA) and TriTanium^®^ (TR) Multiforce Nickel-Titanium Orthodontic Archwires

**DOI:** 10.3390/ma16020483

**Published:** 2023-01-04

**Authors:** Angelina Stoyanova-Ivanova, Mirela Georgieva, Valeri Petrov, Laura Andreeva, Alexander Petkov, Velizar Georgiev

**Affiliations:** 1 G. Nadjakov Institute of Solid State Physics, Bulgarian Academy of Sciences, 72 Tzarigradsko Chaussee, 1784 Sofia, Bulgaria; 2 Faculty of Dental Medicine, Medical University of Sofia, St. G. Sofiiski Blvd., 1431 Sofia, Bulgaria; 3 H. H. Wills Physics Laboratory, University of Bristol, Bristol BS8 1TL, UK

**Keywords:** TriTanium^®^ multiforce archwires, Bio-Active^®^ multiforce archwires, nickel-titanium, mechanical properties, dentistry, orthodontics

## Abstract

Multiforce orthodontic archwires are thermodynamic wires made of nickel-titanium alloy (Ni-Ti). They release biologically tolerable forces along their length, progressively increasing from front to back. The frontal archwires’ segments distribute the weakest force: the premolar, the greater, and the molar, the greatest. The aim of the present study was to determine the influence of clinical use on the mechanical properties of two types of multi-force orthodontic archwires (TriTanium^®^, American orthodontics; Bio-Active^®^, GC) with dimensions of 0.016 × 0.022 inches for periods of up to 8 weeks and over 8 weeks of in-vivo use. A three-point bending test was used, and the data gained is statistically analyzed through a multi-variance comparison Mann-Whitney test. We found that after uses of up to 8 weeks and over 8 weeks, the shape memory effect and superelasticity are preserved, as well as the tendency for differential force release along the length of the archwires is kept.

## 1. Introduction

Fixed orthodontic appliances (brackets) are commonly used, and orthodontic archwires are an indispensable part of the Fixed technique (brackets). By engaging the orthodontic archwire in the bracket slot, activating forces are generated. When the archwire is deactivating, tooth movement and bone remodeling processes take place [1].

Treatment is carried out in stages, and the selection of appropriate archwires contributes to treatment success. An ideal archwire for all stages of fixed appliance treatment has not been developed yet. The initial archwire is the first archwire to be inserted into the fixed appliance at the beginning of the treatment and is used mainly for correcting crowding and minor tooth rotations. Light, continuous forces (also known as optimal forces) are thought to be the most desirable to achieve controlled and predictable tooth movement with minimum harm to the teeth and supporting tissues [2,3,4,5]. Clinically, this means that optimal forces result in the maximum speed of tooth movement with minimal root resorption [6].

The use of Ni-Ti archwires is highly recommended for the initial phase of treatment with fixed appliances. They delivered constant and low forces. Nickel-titanium alloy was first discovered by Buehler in 1960 in the U.S. Naval Ordnance Laboratory in White Oak, Maryland [7,8].

Nitinol has a large number of biomedical applications due to thermoelastic martensitic phase transformation and reverses transformation to parent austenite upon heating (shape memory effect) or upon unloading (superelasticity). Another important property of nitinol is its low-elastic modulus, close-to-natural-bone material, with a compressive strength higher than natural bone material, which makes it an ideal material for biomedical and prosthetic implant applications. In the medical field, nitinol has many applications; for example, it can be used as a guided wire and heart valve tool, can be used in the joining of fractured bones, and can be used as a stent. In dental medicine, Nitinol is used for the production of endodontic instruments for root canal treatment and orthodontic archwires [9,10].

Currently, on the market, there is a wide variety of orthodontic archwires made of Ni-Ti alloy, which has been improved over the years by adding various elements or by high-quality heat treatment methods.

Evans and Durning [11] propose a classification via a chronological stage of discovery of the alloys and structural phase. Stage I: alloys free of nickel and titanium, such as steel and gold (1940–1960); stage II: martensitic stabilized alloys such as the original Nitinol; stage III: superelastic, austenitic-active alloys (Chinese and Japanese arcs, 1980); stage IV: thermoactive, martensitic alloys (Cu-Ni-Ti 1990); stage V: graded thermodynamic alloys (GAC BioForce 1990).

Conventional superelastic archwires have the same mechanical properties along the entire arch length. This is their main disadvantage, since to move different groups of teeth in the oral cavity, forces of different magnitudes are required, depending on the volume of the root surface. Multiforce orthodontic archwires are graded thermodynamic wires. They release biologically tolerable forces along their length, progressively increasing in a front-to-back direction. The frontal archwire segment distributes the weakest force, with the premolar segment being–greater and the molar segment the greatest.

For the first time, Ibe & Segner [12] used a three-point bending test to measure the magnitude of the released forces in three segments of the archwires they studied. They found that Forestadent’s Titanol Multiforce in the incisor region released an average force of 223.8 cN, which increased to 251.1 cN in the molar region.

From the research of the orthodontic literature [13,14,15], we found that the information is insufficient for the multi-force archwires TriTanium^®^ and Bio-Active^®^, used in clinical orthodontic practice (in-vivo use). When choosing an appropriate archwire for a certain stage of orthodontic treatment with a fixed technique, information about the mechanical properties is of the utmost importance for the practitioners. In our previous scientific research, we choose to use Nanoindentation as a testing method for mechanical properties [16,17]. This technique allows simultaneous quantitative measurements of hardness. Nanoindentation as a means of characterising orthodontic archwires is a relatively new technique and was first reported by Alcock et al. [18]. Usually, nanoindentation tests have allowed the measurement of mechanical properties for extremely small volumes of materials or materials with oxide layers. Variations in the results for the nanoindentation and conventional mechanical tests can be attributed to the different material volumes sampled, the different work-hardening levels, and perhaps the effect of the oxide layer on the surface [19]. Gathering data from the literature, we decided to use a three-point bending test for the mechanical characterization of the two types multi-force archwires.

The aim of the present study was to determine the influence of clinical use on the mechanical properties of two types of multi-force orthodontic archwires **(TriTanium^®^, American orthodontics; Bio-Active^®^, GC)** with dimensions of 0.016 × 0.022 inches for periods of up to 8 weeks and over 8 weeks.

## 2. Materials and Methods

### 2.1. Ethics Statement

The clinical procedures were carried out according to the guidelines of the World Medical Association’s Declaration of Helsinki and the Ministry of Health for Good Clinical Practice, and they were informed that consent for testing the archwires was obtained from the patients.

### 2.2. Materials

#### 2.2.1. Selection of the Investigated Archwires

Two types of multi-force orthodontic archwires, TriTanium^®^, (3t) American Orthodontics, Sheboygan, Wisconsin and Bio-Active^®^, TOMY Inc., Tokyo, Japan with dimensions 0.016 × 0.022, were studied.

They were classified into three groups according to the period of clinical use as follows: I group—period-G_0_-clinically unused archwires, II group—period-G_1_-clinically used up to 8 weeks, III group—period-G_2_-clinically used over 8 weeks.

After the archwires’ segmentation into pieces (the method is described below in the Methods section) a total of 36 Bio-Active^®^, TOMY Inc., Japan (BA) and 36 TriTanium^®^, (3t) American Orthodontics, Sheboygan, Wisconsin (TR) specimens were classified as follows: sample from frontal section (frontal segment)—12 pcs.; sample from premolar section (middle segment)—12 pcs.; sample from the molar area (posterior segment)—12 pcs. Each archwire was cut into six equal pieces, with a size of 15 mm. We made the decision to use these sizes because the medio-distal width of the central and lateral incisors is about 15 mm, which is similar to canines, premolars, and molars too. Using the millimeter paper, we marked the exact millimeters on the examined archwires.

#### 2.2.2. Marking Code of the Samples

The first number of codes means the type of investigated archwire—number 1 for “TriTanium^®^” orthodontic archwires; 2—“Bio-Active^®^” orthodontic archwires. The second digit means the period of stay of the archwire in the oral cavity: 0—clinically unused archwires; 1—clinically used up to 8 weeks; 2—clinically used over 8 weeks. The third digit marks the studied segment: 1—studied specimen from the frontal section; 2—from the area of the premolars; 3—from the molar area.

### 2.3. Methods

#### 2.3.1. Disinfection Protocol

Clinically used archwires were taken from patients with fixed appliances (braces) and selected according to the treatment plan. The disinfection measures were applied: Oxygen water 3%, Alcohol 95% and final spraying with Isorapid^®^ spray for 1 min. Finally, the samples were washed under running water to remove the disinfectant solutions. After the disinfection protocol, we proceed to segmentation.

#### 2.3.2. Segmentation of the Archwires

Firstly, with a fine writing marker (0.5 mm), the middle of each archwire in the area of the central incisors is marked. From the middle point, 15 mm was measured bilaterally so as to form 2 frontal segments with a total length of 30 mm.

Secondly, from both ends of the frontal segment, a new 15 mm was measured bilaterally, corresponding to the area of the premolars or premolar segment. Thirdly, for the final molar segment, 15 mms were measured bilaterally.

Fourthly, after marking the areas, we proceeded in cutting the marked places using an orthodontic cutter. The ends of each segment were smoothened with a diamond bur.

#### 2.3.3. Three-Point Bending Test

The three-point bending test follows the recommendations of the ISO 15841 (2014) standard and was carried out at the Faculty of Dental Medicine–Sofia.

The purpose of the examination was to obtain the maximum informativeness of the results of the mechanical tests by simulating the real processes of the interaction between the forces and deformations of the orthodontic archwires after clinical application, which were fixed to the dental arch.

**Figure 1:**
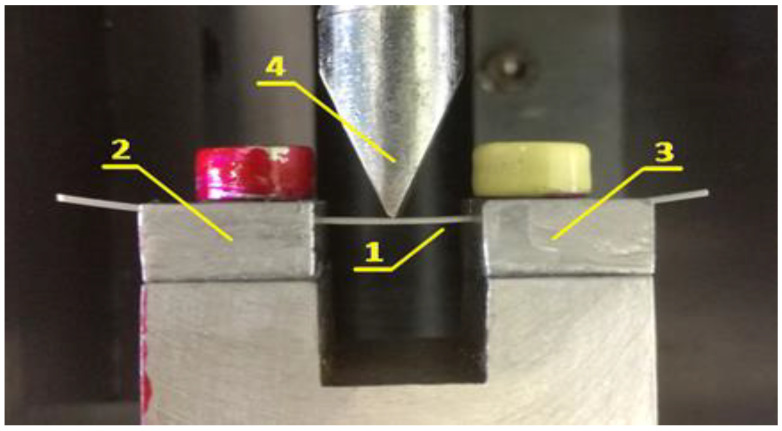
Three-point bending test apparatus. Position 1 indicates the sample being tested; Positions 2 and 3 indicate the specially made incisions in the props; Position 4 indicates the indenter.

A three-point bending test was carried out on a specially made appliance (wire-holder) for better fixing of the wire pieces on the exact side (0.016), without making additional deformations and twisting on the tested segments. It was fixed to a standard LMT-100 (LAM Technologies, Firenze, Italy) for physical-mechanical testing, which is shown in Figure 1.

Position “1” indicates the tested specimen. It rests symmetrically in specially made slots of supports “2” and “3”. The distance between the supports is fixed and is 10 mm. The slots are 0.44 mm wide and 2 mm deep along the entire length of the support. Their form limits the possibility of unwanted deformation processes. In this way, the idea of ”controlled” free contact of the orthodontic archwire with the supports is guaranteed without removing the possibility of slipping during loading.

All tests on the mechanical properties of the archwire were performed at a temperature of 36 °C, which is close to the temperature of the oral cavity. The temperature was regulated using a thermostat, keeping it constant. Measurements of the mechanical properties of each type of archwire were performed also for the three sections formed after segmentation. The unloading forces in the UNLOAD phase were measured at 0.5, 1.0, 1.5, and 2.0 mm, respectively.

The test is carried out on the short side-0.016 inch of the 0.016 × 0.022-inch orthodontic archwires. The measuring units used in the three-point bending test are in N (Newton), or 1 Newton (N) = 100 cN, which is approximately equal to 101.97 g of force (gf).

#### 2.3.4. Statistical Methods

To check the normality of the sample’s distribution Chi-Square Goodness of Fit test is used. The null hypothesis is that the data comes from a normal distribution against the alternative hypothesis that the data does not come from such a distribution. After rejecting the null hypothesis, the non-parametric Kruskal-Wallis one-way ANOVA test is used to obtain test statistics for group comparison. The significance level of the differences between the groups has been determined by the performance of multi-variance comparison based on the ANOVA test statistics, using Mann-Whitney multiple comparative tests (MCT), Bonferroni corrected for group comparison. The results are considered statistically significant if they have reached a confidence level higher than 95% (*p* < 0.05).

This study’s statistical data processing and methods implementation has been performed on MATLAB^®^, (R2020a). Natick, Massachusetts: The MathWorks Inc., (Natick, MA, USA) through custom and system MATLAB^®^ functions.

The three-point bending test is used and the data gained is statistically analyzed through an ANOVA non-parametrical Kruskal Wallis test and a multi-variance comparison Mann-Whitney to reject or accept the hypothesis that the mechanical properties of multiforce archwires does not change after clinical use.

## 3. Results

The obtained results give important information about the changes in the values of the released forces after the clinical application of the researched orthodontic archwires’ in the oral cavity.

Following the results of archwires in group I, for period G_0_, we should be able to report the influence of clinical use on the mechanical properties for groups II and III and periods of use for G_1_ and G_2_.

Table 1 and Table 2 present the summarized average values of the released forces at different deformations (0.5, 1.0, 1.5, 2.0 mm) of the **Bio-Active^®^** and **TriTanium^®^** archwires after conducting a three-point bending test in the three segments at a temperature of 36 °C.

The data, collected from Three-point bending test conducted on **Bio-Active^®^** archwires, which indicates that differential force release is reported. The forces are progressively increasing in the front-to-back direction. At 2 mm deflection, the mean released forces for frontal segments are below 4 N between 3.8 N and 4 N. For the premolar segment, measured mean forces are above 4 N–between 4 N and 4.2 N; the molar segment released force values 5 N. The released forces are in direct ratio to the archwires’ deformation, i.e., a bigger deformation and larger forces are released, as this rule applies to all three segments.

The three-point bending test performed on an unused **TriTanium^®^** archwire and the obtained data show different magnitude force release in the different segments along the entire length of the orthodontic archwire at a 2 mm deviation. The frontal segment shows a force release of 4 N; the premolar segment–forces of 4.5 N; the molar segment-forces between 5 N and 6 N.

**Figure 2:**
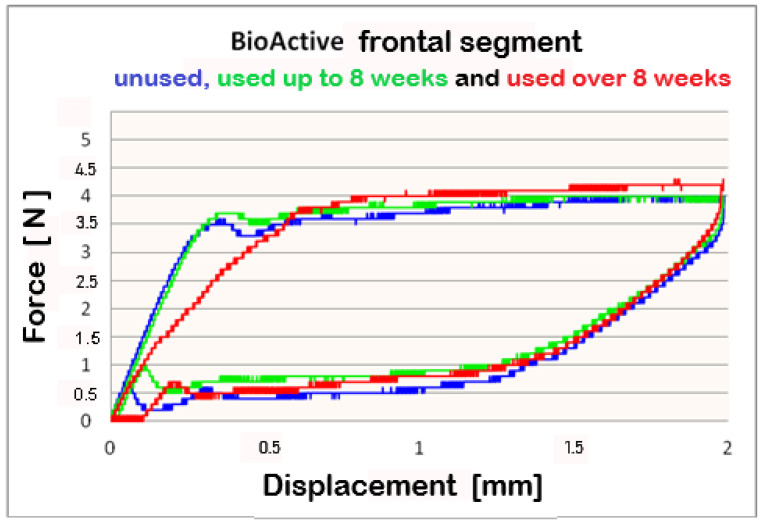
Combined force/deformation curve graphs for period G_0_ (blue), G_1_ (green)_,_ G_2_ (red), frontal segment for the **Bio-Active^®^** archwire.

**Figure 3:**
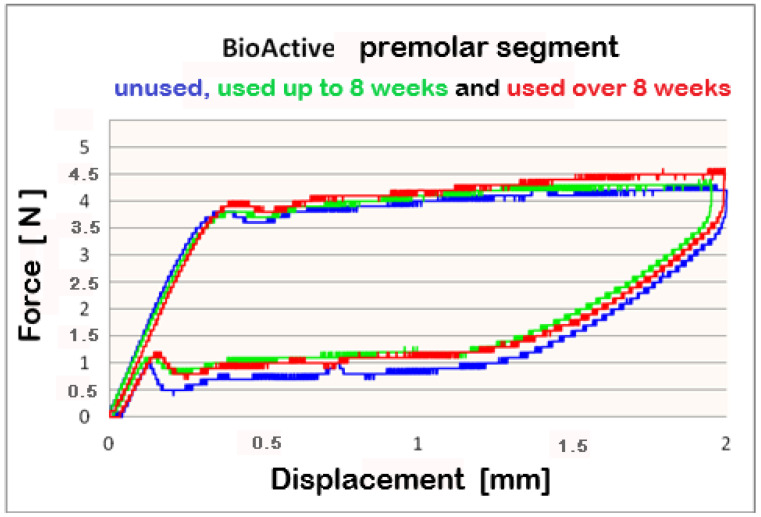
Combined force/deformation curve graphs for period G_0_ (blue), G_1_ (green)_,_ G_2_ (red), premolar segment for the **Bio-Active^®^** archwire.

**Figure 4:**
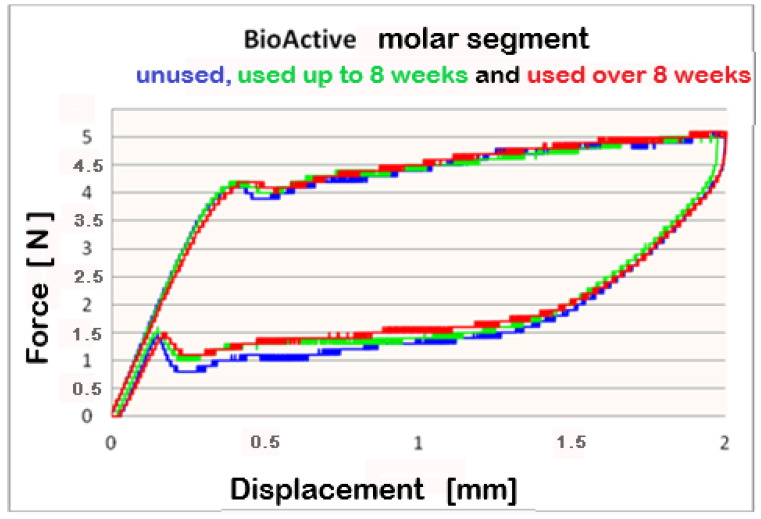
Combined force/deformation curve graphs for period G_0_ (blue), G_1_ (green)_,_ G_2_ (red), molar segment for the **Bio-Active^®^** archwire.

**Figure 5:**
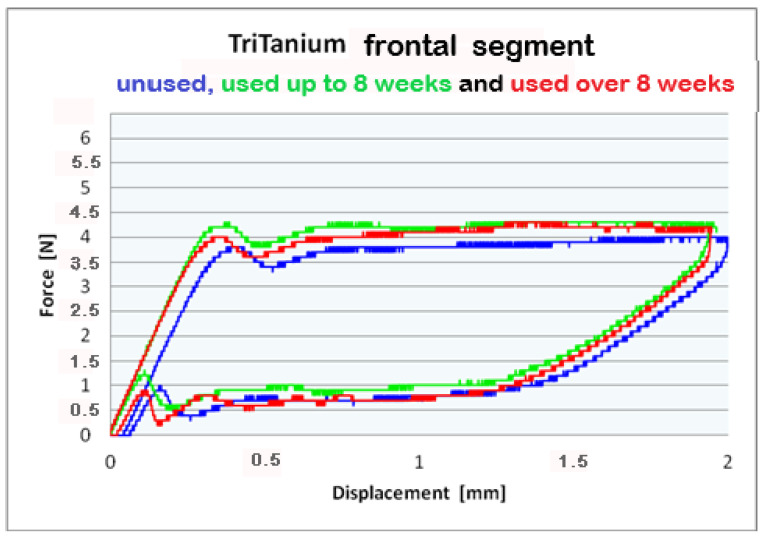
Combined force/deformation curve graphs for period G_0_ (blue), G_1_ (green)_,_ G_2_ (red), frontal segment for the **TriTanium^®^** archwire.

**Figure 6:**
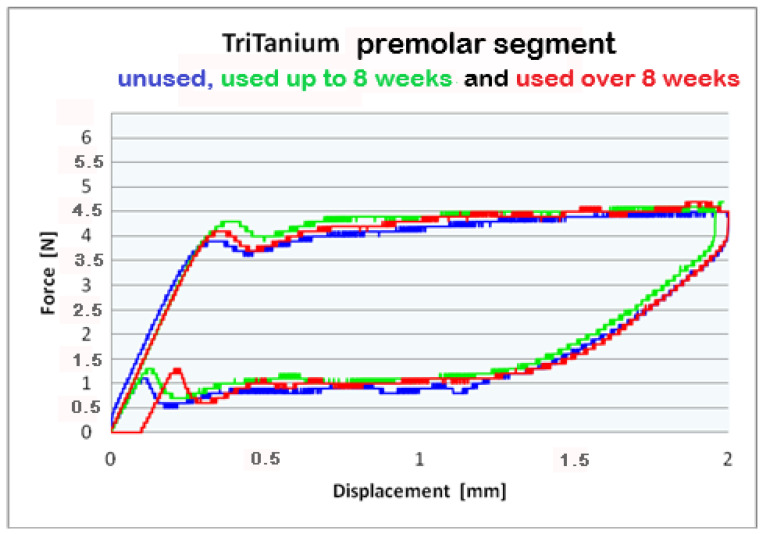
Combined force/deformation curve graphs for period G_0_ (blue), G_1_ (green)_,_ G_2_ (red), premolar segment for the **TriTanium^®^** archwire.

**Figure 7:**
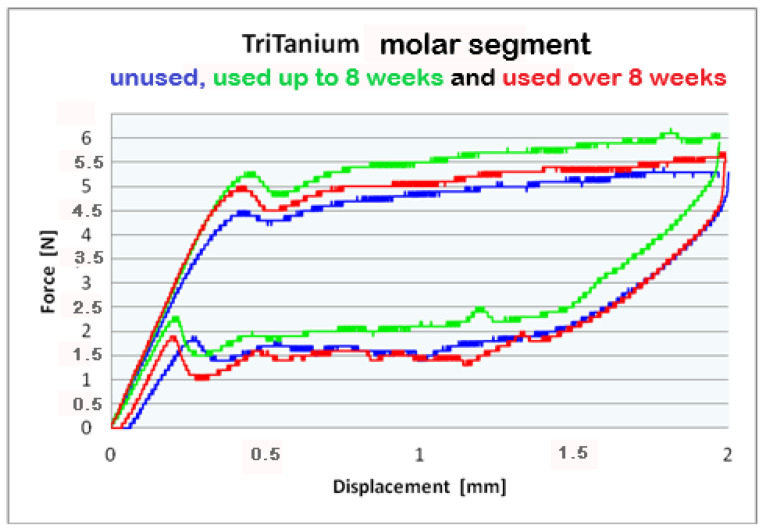
Combined force/deformation curve graphs for period G_0_ (blue), G_1_ (green)_,_ G_2_ (red), molar segment for the **TriTanium^®^** archwire.

For better visualization of the results from the three-point bending test, combined force/deformation curve graphs are presented for groups I, II, and III in the frontal, premolar, and molar segments of the Bio-Active^®^ (Figure 2, Figure 3 and Figure 4) and TriTanium^®^ (Figure 5, Figure 6 and Figure 7) archwire at 36 °C.

After a three-point bending test was performed on each segment (frontal, premolar and molar) of unused Bio-Active^®^ and TriTanium^®^ archwire, which served as our baseline, differential force release along the length of the archwires was reported: the frontal archwires’ segments distribute the weakest force, then the premolar, and finally the molar, which distributes the greatest force. There are differences in the shape of the plateau for the studied segments; the molar segment has better superelastic properties and smaller hysteresis. At a 2 mm displacement of the archwires in the deactivation phase, the released forces are the highest; from 1.5 mm to 0.5 mm deformation, the released deactivation forces are kept nearly constant. Orthodontic graduated thermodynamic archwires possess specific properties, such as “shape memory” effect and superelasticity. As seen from the graphic (groups II and III), these properties are preserved for long periods of clinical use and no plastic deformation was observed in the researched samples.

We found that after a clinical use of up to 8 weeks and over 8 weeks, the shape memory effect and superelasticity are preserved, as well as the tendency for differential force release along the length of the archwires, which is kept during usage. In the three segments of used Bio-Active^®^ orthodontic archwires in group II, in the process of deactivation, the following tendency is again observed, with a decrease in the deformation (controlled deviation); the released forces also decrease. Released forces at 2 mm, 1.5 mm and 0.5 mm deformation have statistically significant differences, being the highest at 2 mm deflection/deformation and the lowest at 0.5 mm deflection.: frontal 4 N (400 cN), premolar 4.3 N (430 cN), molar 5 N (500 cN) at 2 mm deformation; frontal 0.5 N (50 cN), premolar 1 N (100 cN), molar 1.2 N (120 cN) at 0.5 mm deformation. In group III of Bio-Active^®^, the values of forces are as follows: frontal segment 4.2 N (420 cN), premolar 4.4 N (440 cN) and molar 5.2 N (520 cN) at 2 mm and 0.7 N (70 cN) frontal; premolar 1.2 N (120 cN) and molar 1.4 N (140 cN).

Archwires TriTanium^®^ in group II and III during deactivation shows the same tendency with higher values comparing those in Bio-Active^®^: 2 mm deformation delivered forces as follows: frontal segment 3.9 N (390 cN), premolar 4.5 N (450 cN) and molar 5.2 N (520 cN); 0.5 mm deformation of the archwires: frontal 0.6 N (60 cN), premolar 1.0 N (100 cN) and molar 1.4 N (140 cN). Values for group III are as follows: for deformations of 2 mm: a frontal segment of 4.0 N (400 cN), a premolar of 4.4 N (440 cN), and molar of 5.6 N (560 cN); for 0.5 deformations: a frontal of 0.7 N (70 cN), a premolar of 1.0 N (100 cN), and a molar of 1.5 N (150 cN).

**Figure 8:**
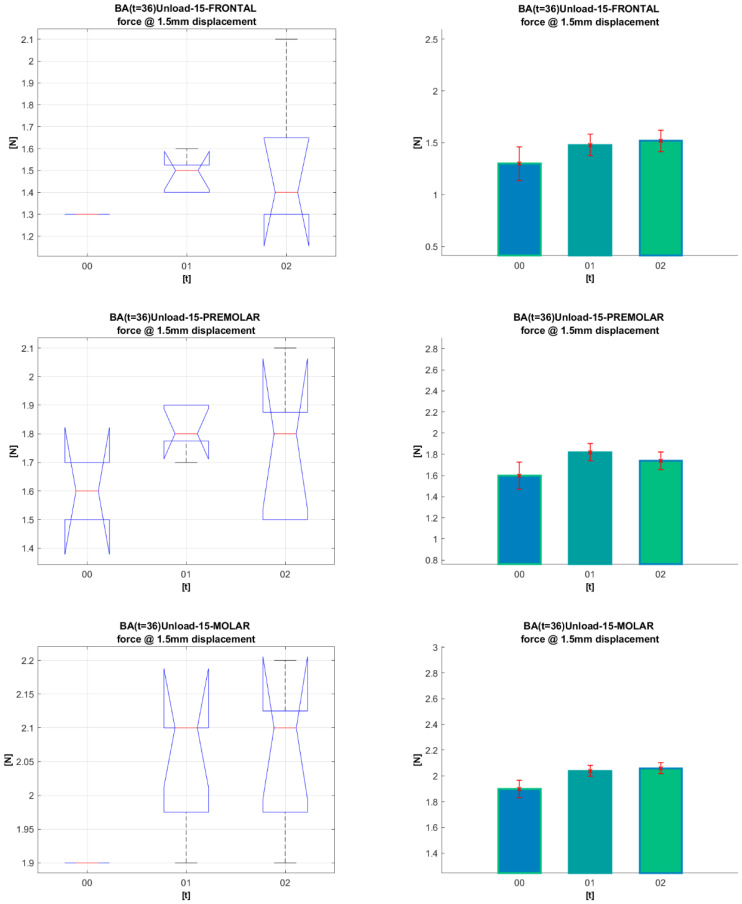
Statistic analysis for 1.5 mm deformation of **Bio-Active^®^** archwires for periods (G_0_, G_1,_ G_2_).

**Figure 9:**
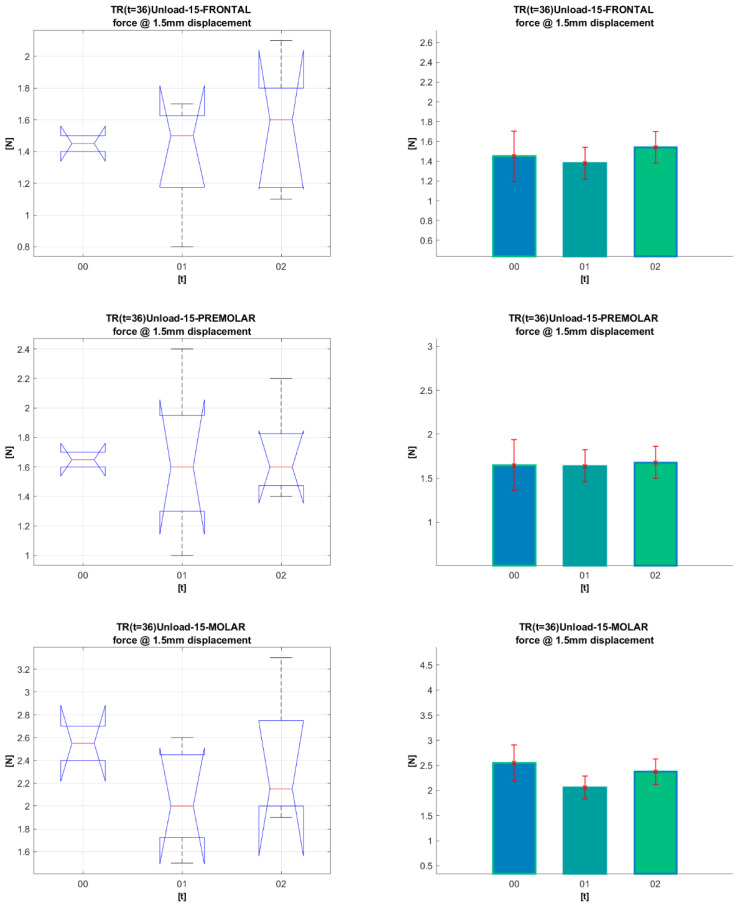
Statistical analysis for 1.5 mm deformation of **TriTanium^®^** archwires for periods (G_0_, G_1_, G_2_).

Figure 8 and Figure 9 present a statistical analysis of the dynamics of the forces for each segment in the G_0_, G_1_, and G_2_ periods of clinical use, at 1.5 mm archwire deformation for Bio-Active^®^ and TriTanium^®^. The same statistical analysis was done for all other deformation values (0.5 mm, 1 mm, and 2 mm). All the *p*-values resulting from the comparisons are given in Table 3 and Table 4 after Figure 8 and Figure 9.

ANOVA box plot shows the graphical results of the ANOVA test (left-side graphics). On each box, the central mark indicates the median, and the bottom and top edges of the box indicate the 25th and 75th percentiles, respectively. The whiskers extend to the most extreme data points not considered outliers, and the outliers are plotted individually using the ‘+’ marker symbol. The notches in the boxes correspond to a 95% confidence interval. Since the notches in the box plot do not overlap, you can conclude, with 95% confidence, that the actual medians do differ.

Based on ANOVA statistics, one may produce a mean bar plot with error bars (right-side graphics). The *X*-axis shows the compared groups. The bars represent the mean of the compared quantification measure for each group (*Y*-axis). The group mean is represented by a bar. Error bars are lines from the top of the bar parallel to the *Y*-axis, representing the uncertainty or SD of the mean value. Error bars communicate how to spread the data around the mean value. A “significant difference” means that the results are likely not due to chance or sampling error. We marked it with aesthetics, which in terms of *p*-values is (* *p* < 0.05; ** *p* < 0.01; *** *p* < 0.001).

The statistical analysis conducted on **TriTanium**^®^ and **Bio-Active^®^** for the three segments (frontal, premolar and molar), for the periods of clinical use (G_0_, G_1_, and G_2_) and archwires’ deformation at 0.5 mm, 1.0 mm, 1.5 mm, and 2.0 mm, did not show statistically significant differences. Comparing the segments (frontal, premolar, and molar) of TriTanium^®^ versus Bio-active^®^ shows that TriTanium^®^ releases slightly higher forces. Nevertheless, after clinical use for periods of up to and over 8 weeks, differential force release along the length of TriTanium^®^ and Bio-Active^®^ archwires is kept as forces are progressively increasing from front to back.

## 4. Discussion

The force in play to align and level the teeth is not the activation force but the deactivation force, or unloading force, of the appliance. The activation and deactivation behavior of a wire might not be the same. Therefore, force/deflection graphs generated during the activation (loading) and deactivation (unloading) cycles of a wire might not superimpose. Hence, knowledge of deactivation behavior is important to the clinician for optimal wire selection [1,20]. Therefore, the linear region corresponding to the deactivation plateau is lower than the activation plateau and parallel to it. This phenomenon is called hysteresis. The main clinical interest of hysteresis is that the force delivered to the periodontal structures is lower than the force necessary to activate the wire [21]. Rygh and Proffit et al. recommend not exceeding a force of 70 cN in the anterior sector to minimize the risk of root resorption [22,23].

In our research, we have focused on the role of deactivating forces in the unloading phase, and we have found that the results obtained in the archwires’ activation phase are logically higher than in the deactivation phase.

A recent study by L. Lombardo et al. [14] performed on several multi-force orthodontic archwires (Ni-Ti Bio-Active GC^®^); Ni-Ti MultiForce Bio-Vis (ACME^®^) and (MultiForce Bio-Vis, ACME^®^) found that the frontal segment released 0.6N(63 cN); the premolar 1.09 N (109 cN) and the molar 1.28 N (128 cN). Sanders et al. [13] compare rectangular multi-force nickel-titanium wires with rectangular wires with one force zone. In their study, they used the Orthodontic Measuring and Simulation System. The Bio-Active^®^ wire shows varying force levels with the lowest forces in the incisor region 0.6 N (60 cN); medium forces in the bicuspid region 1.5 N (150 cN); highest forces in the molar region 2 N (200 cN) at 1mm deflection. In our study, we compare forces of unused multi-force Ni-Ti orthodontic archwires (Bio-Active, GC, TriTanium, AO) with sizes 0.016 × 0.022. For 1 mm deformation from the three-point bending test in the unloading phase, the released forces for Bio-Active^®^ were at the frontal segment 0.5 N (50 cN.), at the premolar region 0.8 N (80 cN) and 1.3 N (132 cN) at the molar segment. The released forces in the deactivation phase at 1 mm deformation for TriTanium^®^ were a frontal segment of 0.8 N (80 cN), a premolar segment of 0.9 N (90 cN), and a molar of 1.7 N (170 cN). These values increased in both archwires for 2 mm deformation. The differences in the values of the mentioned studies are likely due to different methods of examination; however, all studies proved that multi-force archwires release different forces in the three distinct segments. The limitations of our study are that during the movement of the teeth along the archwires, there is friction between the archwires and the slots of the brackets, which may lead to some changes in released forces. Future studies would be conducted on typodonts with fixed braces.

M. Sarul et al. [15] have evaluated the changes in deactivation forces (F_dav_) in the archwires after 4–6 weeks of use and the influence of the oral environment. They investigated three types of initial single-force Ni-Ti archwires (NeoSentalloy, Cu-Ni-Ti 35 °C and Titanol Superelastic), respectively divided into a round (0.016) and rectangular (0.016 × 0.022), used and unused. In the round group, used superelastic archwires (Titanol Superelastic), a statistically significant decrease in deactivation force was reported, but round Thermoactive, clinically used NeoSentalloy, Cu-Ni-Ti 35 °C, showed no change in deactivation force values. A drop-in deactivation force was again reported in the rectangular Titanol Superelastic group, but not in the NeoSentalloy and Cu-Ni-Ti 35 °C heat-activated group. In their study, heat-activated shape memory archwires showed better mechanical properties after clinical use.

From our scientific study, based on an investigation of unused and clinically used archwires with differential force release, the following tendency was found: Bio-Active^®^ orthodontic archwires in group II released higher forces in the deactivation phase compared with those in group I. Force values are kept constant in group II and III. However, this tendency is not confirmed statistically, as there were no statistically significant differences between the three groups. TriTanium^®^ archwires show another type of tendency: an initial increase in the deactivation forces in group II compared with group I and a subsequent decrease in the forces in group III. These results did not show statistically significant differences. In the Wayman study [24], cracks were noted at the edge of the archwire, caused by the presence of tensile forces arising from the engagement of the orthodontic archwires into the brackets. These forces lead to changes in the microstructure of the alloy, including a reduction in the size of the pores in the alloy at the pressure points.

According to Liu D.X., there is a theory [25] where reducing the size and number of pores in the alloy’s structure is directly related to increasing hardness. Due to this, there is a tendency in group II for an initial increase in the released forces.

Measurements for Nickel ions content were made in pores with very probable corrosion processes. A study [26] conducted on the dynamics of nickel content (NC), after different periods of intraoral use of archwires (Ni-Ti, heat-activated Ni-Ti and Cu-Ni-Ti), found that there are statistically significant differences. Ni-Ti and heat-activated Ni-Ti, after the first six to seven weeks of use, the NC significantly decreases.

The formation of Ni-rich precipitates leads to significant changes in mechanical properties. Deformation of the alloy could lead to localized amorphization of the matrix phase around the SPPs [27].

Future studies are needed in order to test chromium and nickel release and also for the wires tested in the present report. Such studies have been done on orthodontic archwires made from stainless steel [28,29], which provide important information.

## 5. Conclusions

- The results show that the released forces and the mechanical properties of the archwires (Bio-Active^®^ and TriTanium^®^) are preserved for a period of over 8 weeks.
- Comparing the results for groups I, II, and III, no statistically significant differences were found.
- This makes their use possible even when regular monthly meetings with patients are impossible.
- With their graduated biologically tolerable forces multi-force archwires are particularly suitable for patients with periodontal problems and little crowding.

## Figures and Tables

**Table 1:** Summarized average values of the released forces of Bio-Active^®^ archwires.

	CODE	LOAD Displacement [mm]					UNLOAD Displacement [mm]				
		0.5	1.0	1.5	2.0		0.5	1.0	1.5	2.0	
FRONT	**201**	3.3	3.6	3.9	3.9		0.3	0.5	1.3	3.9	**FORCE**
						SD	0.2	0.1	0.0	0.0	
	**211**	3.5	3.8	4.0	4.0		0.7	0.9	1.5	4.0	
						SD	0.1	0.1	0.0	0.2	
	**221**	3.6	4.1	4.3	4.2		0.8	1.0	1.7	4.2	
						SD	0.5	0.3	0.4	0.2	

PREMOLAR	**202**	3.6	3.9	4.1	4.1		0.6	0.8	1.6	4.1	
						SD	0.2	0.1	0.1	0.1	
	**212**	3.8	4.2	4.3	4.3		1.1	1.2	1.9	4.3	
						SD	0.1	0.1	0.1	0.1	
	**222**	3.9	4.2	4.4	4.4		1.1	1.3	1.9	4.4	
						SD	0.2	0.2	0.2	0.2	

MOLAR	**203**	3.8	4.4	4.8	5.0		1.1	1.3	1.9	5.0	
						SD	0.1	0.1	0.0	0.0	
	**213**	4.0	4.5	4.8	5.0		1.3	1.5	2.1	5.0	
						SD	0.1	0.1	0.0	0.1	
	**223**	4.2	4.6	5.1	5.2		1.4	1.6	2.1	5.2	
						SD	0.1	0.1	0.1	0.3	

**Table 2:** Summarized average values of the released forces of TriTanium^®^ archwires.

	CODE	LOAD Displacement [mm]					UNLOAD Displacement [mm]				
		0.5	1.0	1.5	2.0		0.5	1.0	1.5	2.0	
FRONT	**101**	3.5	3.9	4.0	4.0		0.7	0.8	1.5	4.0	**FORCE**
						SD	0.0	0.0	0.1	0.1	
	**111**	3.7	4.1	4.2	4.1		0.8	0.8	1.6	4.1	
						SD	0.1	0.2	0.1	0.1	
	**121**	3.6	4.0	4.0	3.9		0.5	0.7	1.5	3.9	
						SD	0.3	0.3	0.3	0.3	

PREMOLAR	**102**	3.8	4.2	4.4	4.5		0.8	0.9	1.6	4.5	
						SD	0.0	0.0	0.0	0.1	
	**112**	4.1	4.6	4.8	4.8		1.1	1.3	1.9	4.8	
						SD	0.3	0.4	0.4	0.5	
	**122**	3.6	4.1	4.3	4.3		0.7	0.9	1.6	4.3	
						SD	0.3	0.3	0.1	0.4	

MOLAR	**103**	4.4	5.0	5.3	5.6		1.6	1.7	2.5	5.6	
						SD	0.1	0.1	0.2	0.4	
	**113**	4.5	5.0	5.2	5.3		1.5	1.6	2.3	5.3	
						SD	0.5	0.5	0.4	0.8	
	**123**	4.3	4.9	5.4	5.4		1.4	1.4	2.1	5.4	
						SD	0.3	0.3	0.2	0.6	

**Table 3:** Bio-Active^®^ *p*-values, resulting from the statistical comparison of each segment in the G_0_, G_1_, G_2_ periods of clinical use, at {0.5, 1.0, 1.5, 2.0} mm deformation.

*p-Values Bio-Active^®^*		Displacements			
Segments	Groups	0.5	1.0	1.5	2.0
Frontal	G_0_–G_1_	0.99357	0.69191	1	1
	G_0_–G_2_	0.667518	0.69191	0.847142	0.967188
	G_1_–G_2_	1	1	1	1
Pre-molar	G_0_–G_1_	0.214609	0.379306	0.550995	1
	G_0_–G_2_	0.489692	0.68973	1	1
	G_1_–G_2_	1	1	1	1
Molar	G_0_–G_1_	1	0.919958	0.35192	1
	G_0_–G_2_	0.430823	0.341768	0.237375	1
	G_1_–G_2_	0.687607	1	1	1

**Table 4:** TriTanium^®^ *p*-values, resulting from the statistical comparison of each segment in the G_0_, G_1_, G_2_ periods of clinical use, at {0.5, 1.0, 1.5, 2.0} mm deformation.

*p-Values TriTanium^®^*		Displacements			
Segments	Groups	0.5	1.0	1.5	2.0
Frontal	G_0_–G_1_	1	1	1	1
	G_0_–G_2_	1	1	1	1
	G_1_–G_2_	1	1	1	1
Pre-molar	G_0_–G_1_	1	1	1	1
	G_0_–G_2_	1	1	1	1
	G_1_–G_2_	1	1	1	1
Molar	G_0_–G_1_	1	1	0.833637	1
	G_0_–G_2_	1	1	1	1
	G_1_–G_2_	0.980193	1	1	1

## Data Availability

The datasets used and/or analyzed during the current study are available from the authors on reasonable request.
